# Removal of Organic Dyes from Aqueous Solutions by Activated Carbons Prepared from Residue of Supercritical Extraction of Marigold

**DOI:** 10.3390/ma15103655

**Published:** 2022-05-20

**Authors:** Aleksandra Bazan-Wozniak, Robert Wolski, Dorota Paluch, Piotr Nowicki, Robert Pietrzak

**Affiliations:** Faculty of Chemistry, Adam Mickiewicz University in Poznań, Uniwersytetu Poznańskiego 8, 61-614 Poznań, Poland; aleksandra.bazan@amu.edu.pl (A.B.-W.); wola@amu.edu.pl (R.W.); dorpal1@st.amu.edu.pl (D.P.); piotrnow@amu.edu.pl (P.N.)

**Keywords:** marigold, activated carbons, physical activation, organic dyes, adsorption isotherms

## Abstract

In the present work, we reported on the efficiency of the removal of organic dyes by adsorption on activated carbons prepared from the residue of supercritical extraction of marigold. The performance of adsorbents prepared was tested towards methyl red, methylene blue, malachite green, and crystal violet at room temperature. The effects of carbonization (500 and 700 °C) and activation (700 and 800 °C) temperatures, textural parameters, and acid-base character of the adsorbent surface on the sorption properties of the activated carbons were established. Activated carbons are characterized by low developed specific surface area, from 2 to 206 m^2^/g, and have a basic character of the surface (pH of carbons water extracts ranging from 10.4 to 11.2). Equilibrium adsorption isotherms were investigated. The equilibrium data were analyzed in the Langmuir, Freundlich, and Temkin models. The adsorption capacities of activated carbons studied varied from 47.62 to 102.43 mg/g towards methyl red, 53.14 to 139.72 mg/g towards methyl red, 425.46 to 622.80 towards malachite green and 155.91 to 293.75 mg/g towards crystal violet, from their water solutions. Kinetics of the adsorption of the organic dyes studied were found to be described by the pseudo-second-order model. It was proven that through the physical activation of the residue of supercritical extraction of marigold, it is possible to obtain carbonaceous materials of very high adsorption capacity towards organic pollutants.

## 1. Introduction

It has been established beyond doubt that surface water, including that used for drinking, is polluted by post-industrial and municipal wastewater [1]. The main group of pollutants found in wastewater are organic dyes dispersed in the form of suspensions or dissolved. The majority of them are highly toxic and mutagenic. According to literature data, the global production of about 10,000 organic dyes and pigments reaches 7 × 10^5^ tons, of which 10–15% are released into sewage. The presence of dyes in surface and ground waters, even in trace amounts (less than 1 mg/dm^3^), is strongly undesirable [2].

Methylene blue is a derivative of thiazine and belongs to the most commonly used dyes of wood [3]. Methyl red is widely used in the textile industry and in laboratories [4]. Malachite green represents a group of triphenylmethane dyes and is known to be highly toxic towards mammals’ cells. Crystal violet is a cationic protein dye of known cancerogenic properties. Although its toxicity is unquestionable, it is widely used as bactericidal or antifungal agent [5,6].

A wide gamut of dyes used in many branches of industry that end up in the environment has forced the search for methods of their removal. Adsorption processes have been found effective for such tasks [7,8] and, from among them, particularly useful are those based on activated carbons [9,10,11,12]. The current adsorbents should also show high effectiveness of adsorption of a wide range of adsorbates at the same time. The research groups should be presently concerned with obtaining “intelligent adsorbents” that would permit simultaneous removal of a number of pollutants, e.g., organic dyes of different structures and properties. Thus, the future adsorbents should be more universal and the cost of the adsorption processes should be reduced.

Activated carbons are universal adsorbents for the removal of organic and inorganic pollutants from wastewater and drinking water. Carbon sorbents are usually obtained from the natural precursors of organic origin such as bituminous coals, brown coals, wood, cellulose, peat, fruit stones, and peels [13,14,15] in the processes of physical or chemical activation [15]. Physical activation is a much cheaper process leading to effective carbon adsorbents, so in the studies reported in this paper, we were concerned with carbon adsorbents obtained by physical activation. In near future a new and interesting group of carbon precursors can be the waste of supercritical extraction of plants with CO_2_ [16]. The number of publications on the use of supercritical extraction for obtaining natural substances from herbal plants has been increasing each subsequent year. Moreover, supercritical CO_2_ extraction belongs to the so-called “green technologies”, so its use is undoubtedly beneficial for the image of firms that use this process. One of the plants used as raw products in the process is marigold that is characterized by rich and diverse composition. Marigold flowers are an abundant source of extracts for medical use. The extracts obtained in supercritical conditions are high quality products containing exclusively the biologically active components of the plant and enjoy growing popularity. At present, the residue of supercritical extraction of marigold flowers is just a problematic waste that can be either added to animal forage or combusted, so its profitable utilization is a definite advantage [17,18].

One of the possible ways of its utilization, alternative to combustion, is conversion into activated carbon adsorbents capable of removing pollutants from liquid or gas phases.

The aim of this study was to obtain and characterize a series of activated carbon materials by physical activation of the residue of supercritical extraction of marigold. The process of activation was realized in two stages: carbonization and activation with carbon dioxide. The sorption capacities of the obtained activated carbons were characterized towards four organic dyes: methyl red, methylene blue, malachite green, and crystal violet. The obtained results were analyzed assuming three models proposed by Langmuir, Freundlich and Temkin. The effects of the temperatures of carbonization and activation processes as well as the physicochemical properties of the activated carbons on their sorption capacities were determined. The results obtained in this study were compared with relevant literature data. Taking into account the outcome of our earlier studies described in [16,19], concerning the carbon adsorbents obtained by chemical activation of the residues of supercritical CO_2_ extractions of plant raw products, we decided to apply in this study the method of physical activation to reduce the cost of the adsorbents production. Moreover, the process of carbonization was carried out at 500 °C, so lower than that proposed in [20], which was chosen also to reduce the cost of biocarbon adsorbents production.

## 2. Materials and Methods

### 2.1. Chemicals Reagents

All organic dyes: methyl red, methylene blue, malachite green, and crystal violet used as model adsorbates were purchased from the Avantor Performance Materials Poland S.A. (Gliwice, Poland). These reagents were bought in high purity and were used without further purification.

### 2.2. Preparation of Activated Carbons

The starting material-residue of supercritical extraction of marigold (P) with particle size range of 0.10–0.75 mm, moisture content of 4.9 wt. % and ash content of 9.0 wt. % was subjected to carbonization process (C) at 500 (C5) or 700 °C (C7) followed by physical (also called thermal) activation with carbon dioxide (A) at 700 (A7) or 800 °C (A8), according to the procedure described in detail in our earlier work [21].

### 2.3. Adsorption of Dyes

The dyes studied in this work were: methyl red, methylene blue, malachite green and crystal violet. Adsorption of the dyes was performed using the following procedure. Samples of the prepared activated carbons of 25 mg with the particle size 0.09 mm were placed in 100 mL Erlenmeyer flasks and mixed with 50 mL of dye solution with initial concentrations in the range from 5 to 330 mg/L (methyl red (MR) 5–60 mg/L, methylene blue (MB) 5–80 mg/L, malachite green (MG) 5–330 mg/L and crystal violet (CV) 5–160 mg/L) and the suspension was stirred to reach equilibrium, for 24 h (at 22 ± 2 °C). After the adsorption equilibrium had been achieved, the solution was separated from the sorbent by centrifugation at 6000 rpm for 3 min. Measurements were performed with a Frontiner ™ centrifuge FC5515 (OHAUS, Parsippany, USA). The concentration of the dye solution was determined using a double beam UV-Vis spectrophotometer, model Carry 100 Bio (Agilent, Santa Clara, CA, USA). Two parallel series of measurements were made and the results presented are the arithmetic means of those obtained in each series. 

The adsorption capacity was calculated as:

(1) $$q_{e}=\frac{C_{0}-C_{e}}{m}\cdot V$$

where: C_0_—initial dye concentration [mg/L]; C_e_—equilibrium dye concentration [mg/L]; m—weight of the activated carbon [g]; V—volume of the organic dye solution (L) [21].

The effectiveness of removal (R) of the dyes from their water solutions was calculated from the equation [22]:

(2) $$R=\frac{C_{0}-C_{e}}{C_{0}}\cdot 100\;\%$$

To improve the clarity of data presentation, the values of R presented for the lowest and the highest concentrations of the dye solvent used for flooding of the activated carbon.

The effect of the contact time of adsorbate-adsorbent (0–300 min) on the sorption capacity of the activated carbons obtained was investigated in detail. Samples (25 mg) were stirred with 50 mL of solution containing dyes (methyl red 30 mg/L, methylene blue 30 mg/L, malachite green 200 mg/L and crystal violet 90 mg/L) at intervals of 10, 20, 30, 40, 50, 60, 90, 120, 150, 180, 210, 240, 270 and 300 min. The effect of the pH of the organic dye solution on the sorption capacities of the activated carbons obtained was determined as well. The measurements were performed in the range of pH from 3 to 12. The pH was measured using CP-401 pH-meter (ELMETRON, Zabrze, Poland).

### 2.4. Adsorption Isotherms

The adsorption isotherm shows the mechanism of interaction between adsorbate molecules and adsorbent. One of the basic equations describing adsorption isotherm is the Langmuir equation [23]. Langmuir assumed that on the surface of the adsorbent there is a specific number of adsorption centers, each of which is able to adsorb only one molecule. The linear form of the Langmuir isotherm equation is expressed as:

(3) $$\frac{C_{e}}{q_{e}}=\frac{1}{K_{L}\times q_{\max}}+\frac{C_{e}}{q_{\max}}$$

where: C_e_ is the equilibrium concentration [mg/L], q_e_ is the equilibrium adsorption amount [mg/g], K_L_ is the Langmuir adsorption equilibrium constant [L/mg] and q_max_ is the maximum adsorption capacity of the adsorbent [mg/g].

The important features of the Langmuir isotherm may be expressed in terms of the dimensionless constant separation factor R_L_ [16]:

(4) $$R_{L}=\frac{1}{1+K_{L}\times C_{0}}$$

For the favorable sorption, the value of this parameter varies between 0 and 1; for the unfavorable sorption it exceeds 1; for the linear sorption R_L_ is equal 1 and for the irreversible sorption, R_L_ = 0.

The Freundlich isotherm is described by the empirical equation derived assuming that the adsorbent surface is heterogeneous. The logarithmic form of the Freundlich [24] adsorption can be expressed as:

(5) $${\mathrm{logq}}_{e}={\mathrm{logK}}_{F}+\frac{1}{n}{\mathrm{logC}}_{e}$$

where q_e_ is the amount of the dye adsorbed at equilibrium [mg/g], K_F_ is the Freundlich equilibrium constant [mg/g (mg/L)^1/n^], 1/n is the intensity of adsorption and C_e_ is the equilibrium concentration of the dye [mg/L].

According to the Temkin model, the heat of adsorption decreases linearly with increasing surface coverage [25,26].

(6) $$q_{e}={\mathrm{BlnA}}_{T}+{\mathrm{BlnC}}_{e}$$

where q_e_ is the amount of the dye adsorbed at equilibrium [mg/g], C_e_ is the equilibrium concentration of the dye [mg/L], B constant relates to the heat of adsorption and is expressed in terms of B = RT/B_T_, B_T_ is the Temkin constant [J/mol], R stands for the gas constant (8.314 J/mol K), A_T_ Temkin isotherm equilibrium binding constant [L/mg].

### 2.5. Adsorption Modeling

In order to investigate the kinetics of organic dyes adsorption on activated carbons, the pseudo-first and the pseudo-second order models were applied to experimental data [16,25]. The linear equation of the pseudo-first-order kinetic model takes the following form:

(7) $$\log\left(q_{e}-q_{t}\right)={\mathrm{logq}}_{e}-\frac{k_{1}}{2.303}t$$

where: q_e_, q_t_—is the amount of the adsorbed dye [mg/g], t—time [min], k_1_—is the pseudo-first order adsorption constant [1/min].

The pseudo-second-order model can be expressed by the following linear form:

(8) $$\frac{t}{q_{t}}=\frac{1}{k_{2}q_{e}{}^{2}}+\frac{t}{q_{e}}$$

where: k_2_ is the second order reaction rate equilibrium constant [g/mg·min].

## 3. Results and Discussion

### 3.1. Adsorption of Dyes on the Activated Carbons

The specific surface area of the obtained activated carbons varies in the range from2 to 206 m^2^/g. The textural parameters of the activated carbons were found to significantly depend on the activation temperature of the carbonizates. The porous structure of the carbon materials obtained includes a large fraction of mesopores, as follows from the average pore diameter determined to vary from 3.63 to 31.78 nm. The number of oxygen groups on the surface of the activated carbons was estimated by the Boehm method. According to the above results, only basic oxygen groups occur on the surface of the carbon materials obtained and their content varies from 5.51 to 8.37 mmol/g. The pH of the water extracts from the activated carbons ranges from 10.4 to 11.2. Detail physicochemical characterization of the carbon materials studied is given in our earlier work [21].

Analysis of the data presented in Table 1 shows that the textural parameters of the activated carbons obtained from the residue of critical CO_2_ extraction of marigold are close to those of the adsorbents obtained from the residue of extraction of chamomile [27] and hops [9]. The carbon sample labeled as PC5A8, showing the best textural parameters from among the samples obtained in this study, still has much lower specific surface area and total pore volume values than the carbons obtained from Sapelli sawdust [28] and coconut shells [29], however, it should be noted that the latter carbon sample was obtained by chemical activation with H_3_PO_4_, so the cost of its production was much higher than that of PC5A8. Unfortunately, our sample PC5A8 does not stand a comparison with the commercial carbon Norit^®^ SX ULTRA [30] whose specific surface area is 1092 m^2^/g so 5 times larger than that of our sample.

Table 2, Table 3, Table 4 and Table 5 present the sorption capacities of the four obtained adsorbents towards the four studied organic dyes. The sorption capacity of sample PC5A7 (Table 2) towards methyl red reaches only 48.95 mg/g, while towards methylene blue—56.36 mg/g. Much greater sorption capacities of the same adsorbent were obtained towards malachite green (495.04 mg/g) and crystal violet (180.68 mg/g). According to the data presented in Table 2, the effectiveness of removal of all dyes decreases with increasing the initial concentration of the dye in solution. The greatest decrease in the adsorption capacity was noted for methyl red, while the lowest—for malachite green. The activated carbons obtained when the carbonization temperature was increased by 200 °C showed lower sorption capacity and lower effectiveness of removal of all dyes than sample PC5A7 (Table 3). However, it should be emphasized that for malachite green and crystal violet the decrease in sorption capacity were far greater than for the other two dyes. As follows from the analysis of the data collected in Table 4 and Table 5, the activation at 800 °C leads to carbon adsorbents characterized by the much greater efficiency of removal of the dyes studied from their solutions than that observed for samples PC5A7 and PC7A7. Moreover, for samples PC5A8 and PC7A8, the highest percentage of dye removal was observed, which refers particularly to methyl red and methylene blue.

The most effective adsorbent was sample PC5A8 obtained by activation of the carbonizate PC5 at 800 °C. Most probably, it is a consequence of the much larger surface area (S_BET_ = 206 m^2^/g) and much better developed porous structure of this sample (V_total_ = 0.118 cm^3^/g) [21] in comparison to the corresponding values obtained for the other activated carbon samples. The adsorption capacity of PC5A8 towards methyl red was 102.43 mg/g, towards methylene blue 139.72 mg/g, towards malachite green 622.80 mg/g and towards crystal violet 293.75 mg/g. The samples activated at 700 °C show much lower effectiveness in removal of the organic dyes studied from water solutions. Most probably the reason is their low surface area, not exceeding 4 m^2^/g. The least effective was sample PC7A7.

Analysis of the data displayed in Table 2, Table 3, Table 4 and Table 5 makes it clear that much poorer results were obtained for adsorption of methyl red and methylene blue. The amount of these dyes adsorbed on the activated carbons was much smaller than that of malachite green and crystal violet. Nevertheless, for all organic dyes more effective adsorbents were samples PC5A8 and PC7A8. The poorer effectiveness of adsorption of methyl red and methylene blue may have stemmed from the size of their molecules. The malachite green and crystal violet have higher molecular masses, they were adsorbed faster and mainly on the surface of the activated carbon, while the smaller molecules of methyl red and methylene blue could have accumulated inside the adsorbent pores.

**Figure 1:**
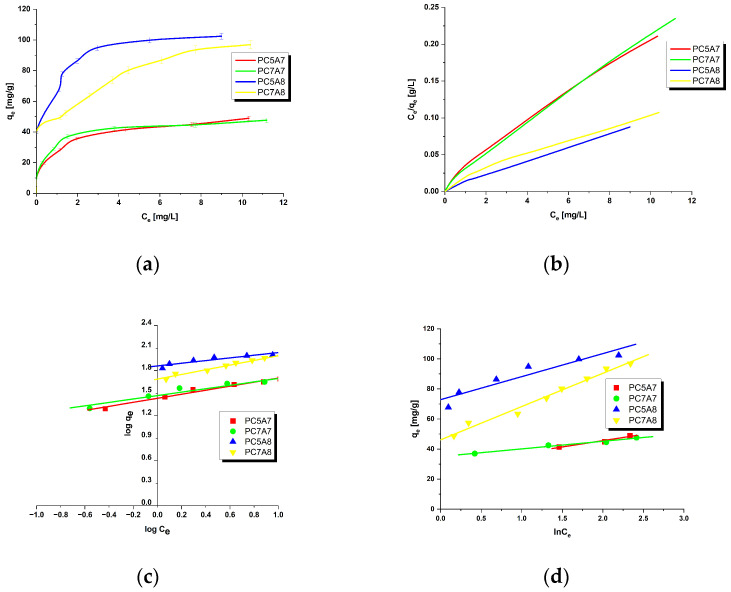
Adsorption of methyl red onto activated carbons (**a**) equilibrium adsorption of the dye, (**b**) Langmuir isotherm plots, (**c**) Freundlich isotherm plots and (**d**) Temkin isotherm plots.

**Figure 2:**
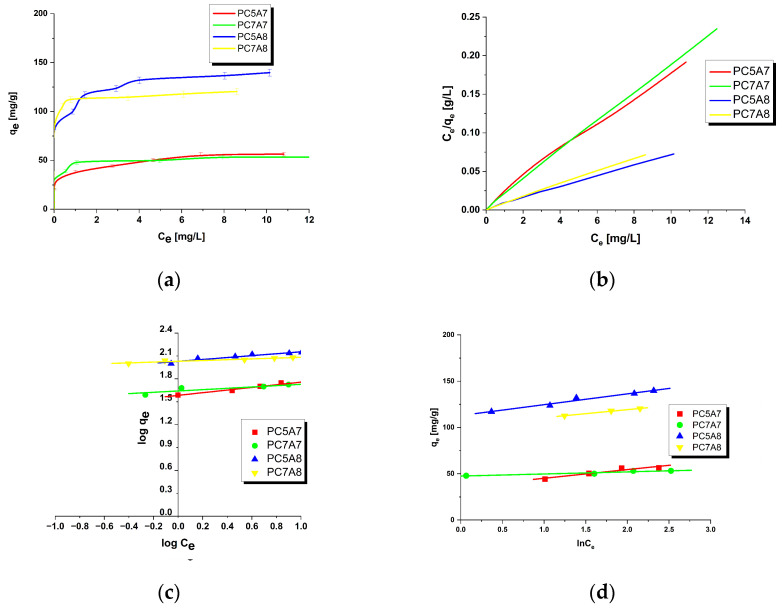
Adsorption of methylene blue onto activated carbons (**a**) equilibrium adsorption of the dye, (**b**) Langmuir isotherm plots, (**c**) Freundlich isotherm plots and (**d**) Temkin isotherm plots.

**Figure 3:**
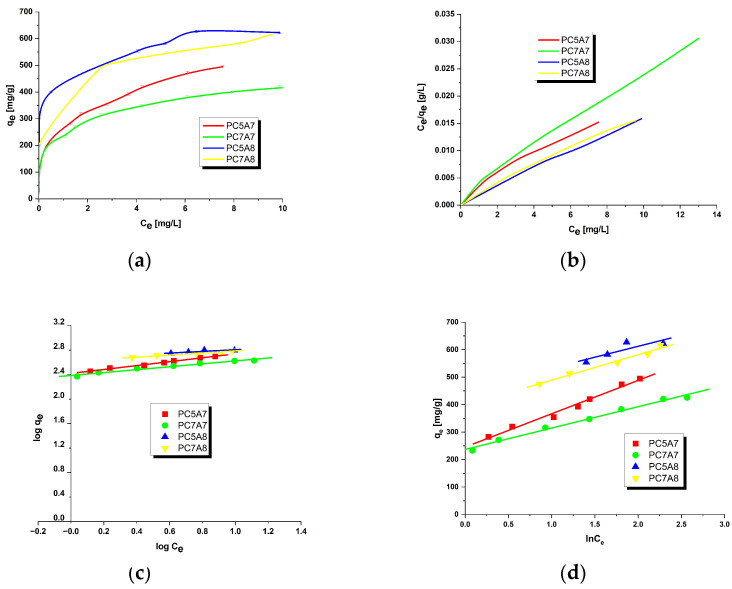
Adsorption of malachite green onto activated carbons (**a**) equilibrium adsorption of the dye, (**b**) Langmuir isotherm plots, (**c**) Freundlich isotherm plots and (**d**) Temkin isotherm plots.

**Figure 4:**
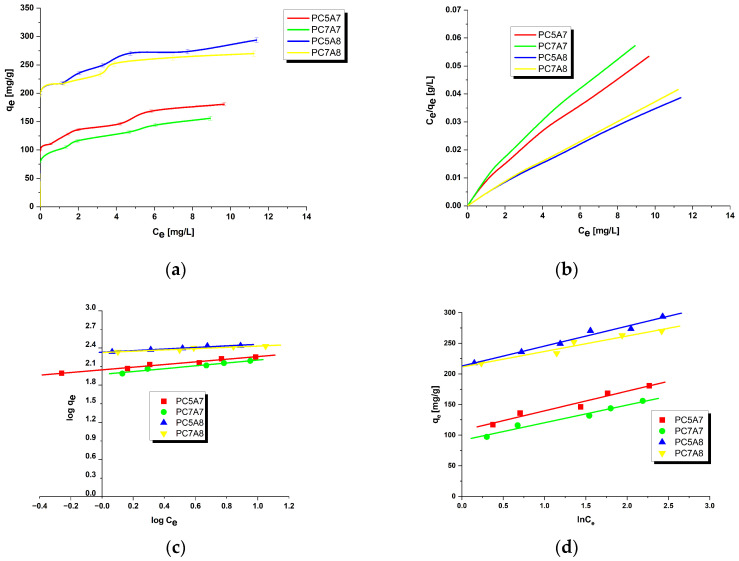
Adsorption of crystal violet onto activated carbons (**a**) equilibrium adsorption of the dye, (**b**) Langmuir isotherm plots, (**c**) Freundlich isotherm plots and (**d**) Temkin isotherm plots.

Figure 1, Figure 2, Figure 3 and Figure 4 present the equilibrium isotherms of the dyes adsorption on the activated carbon samples obtained. The adsorption was measured in mg of the dye per g of adsorbent at 22 ± 2 °C. Analysis of the isotherms indicates that the amount of the dyes adsorbed on the carbon samples significantly increases with an increasing initial concentration of the dye in the solution. This observation can be explained by the fact that at low concentrations of the dyes their adsorption on the activated carbon surface is accidental. At the higher dye concentrations, the active centers on the surface of the activated carbon samples are fully occupied and the surface and/or the porous structure of the adsorbent is saturated. On the basis of the character of adsorption isotherms it is possible to conclude about the mechanisms of interactions between the adsorbate and the adsorbent. A number of models have been proposed for describing the adsorbate-adsorbent interactions. We decided to check the applicability of the three most popular models proposed by Langmuir, Freundlich and Temkin. The parameters characteristics of the models are given in Table 2, Table 3, Table 4 and Table 5, while the courses of Langmuir, Freundlich and Temkin isotherms are presented in Figure 1, Figure 2, Figure 3 and Figure 4.

As follows from the data included in Table 2, Table 3, Table 4 and Table 5, the experimental maximum adsorption capacity (q_e_) for the majority of carbon samples was close to the theoretically calculated value of q_max_. Moreover, close to 1 values of the correlation coefficient R^2^ confirmed a good fit of the Langmuir isotherm to the experimental data, for all organic dyes studied. Therefore, it is reasonable to expect that the dyes adsorption on the activated carbons involves formation of a monolayer on the adsorbate surface [16,31]. The R_L_ values in the range of 0–1 for all samples indicate that the adsorption of organic dyes was favorable. Moreover, the decrease in R_L_ with increasing initial concentration of dyes in solutions indicates that the adsorption was more favorable at higher concentrations.

On the basis of the values of parameter n of the Freundlich model, see Table 2, Table 3, Table 4 and Table 5, it can be concluded that the process of adsorption of all dyes studied was favorable. It is assumed that n in the range 2–10 represents good sorption characteristics, its value between 1 and 2 moderately difficult ones and less than 1 poor ones [13]. For the activated carbon samples obtained from the residue of supercritical extraction of marigold, the value of parameter n varied from 3.2144 to 24.5700, which suggests the prevalent physical adsorption of the dyes. It is assumed that adsorption has chemical character for n < 1, physical character for n > 1 and linear character for n = 1 [13,16]. Additionally, the slope 1/n ranging between 0 and 1, is a measure of adsorption intensity or surface heterogeneity; the surface becomes more heterogeneous as its value gets closer to zero. For the carbon samples studied this value varied from 0.0407 to 0.3111, which indicates a high heterogeneity of their surfaces. Moreover, a value of 1/n below 1 indicates also a normal Langmuir isotherm [13,16,31].

**Figure 5:**
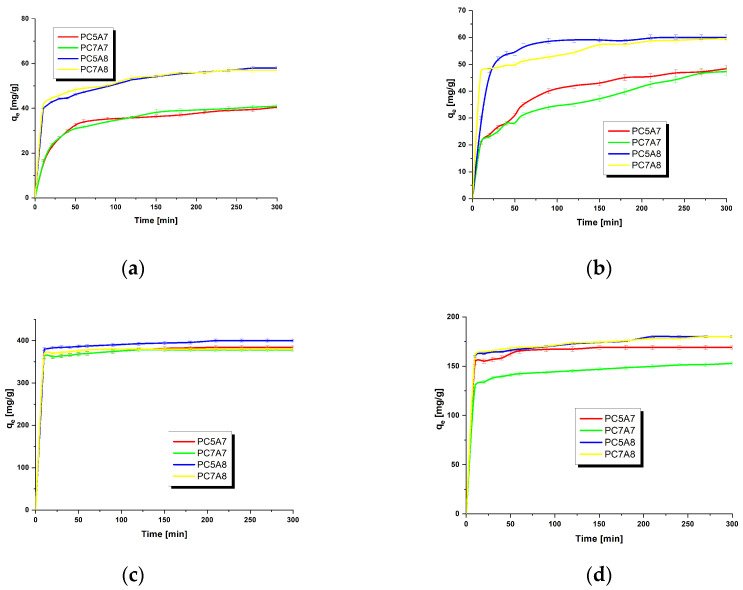
The effect of contact time on the amount of organic dyes adsorbed on the activated carbons: adsorbent mass = 25 mg, volume of solution = 50 mL, temperature =22 ± 2 °C, (**a**) methyl red 30 mg/L, (**b**) methylene blue 30 mg/L, (**c**) malachite green 200 mg/L and (**d**) crystal violet 90 mg/L).

The next task was to characterize the effect of contact time of the organic dye solvent with the adsorbent on the effectiveness of adsorption. The results are displayed in Figure 5. On the basis of the course of the isotherms, it can be concluded that at the beginning of the adsorption process the removal of the dye molecules was fast, most probably because of a large number of free active sites on the adsorbent surface. With increasing contact time, the number of available active sites decreases due to gradual saturation and moreover, the free active sites become less available to the dye molecules because of the repulsive interaction between the adsorbed dye molecules [16]. The state of adsorption equilibrium was reached the fastest for adsorption of malachite green and crystal violet from their water solutions.

**Figure 6:**
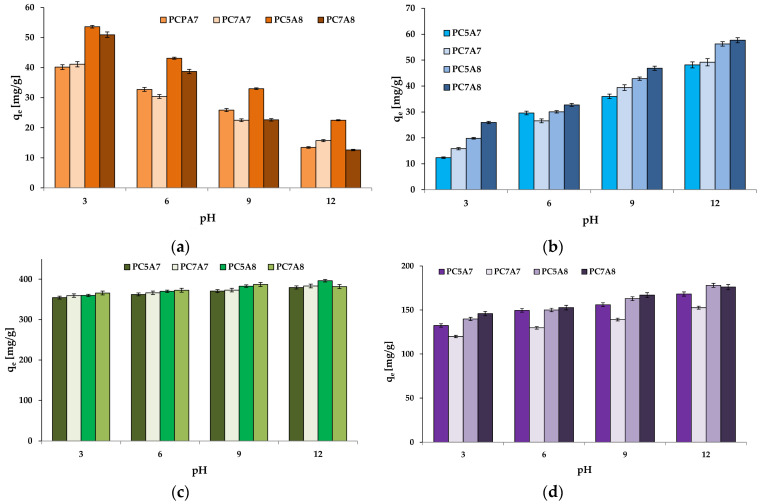
The effect of pH on the amount of organic dyes adsorbed on activated carbons: adsorbent mass = 25 mg, volume of solution = 50 mL, temperature =22 ± 2 °C, (**a**) methyl red 30 mg/L, (**b**) methylene blue 30 mg/L, (**c**) malachite green 200 mg/L and (**d**) crystal violet 90 mg/L).

Another objective of our study was to check the effect of pH of the organic dye solutions on the sorption capacities of the obtained activated carbons. The results for the pH of the dye solution changed from 3 to 12, are presented in Figure 6. As follows from these data, the effectiveness of methyl red removal decreased with an increasing pH of its solution. For the other organic dyes studied, the opposite relation was observed. Analysis of literature data indicates that at low pH, the percentage of removal of an anionic dye increases, while at high pH it decreases [25,31]. The impact of pH of the dye solution was particularly well seen for the adsorption of methyl red and methylene blue from their water solutions on the carbon adsorbents studied.

**Figure 7:**
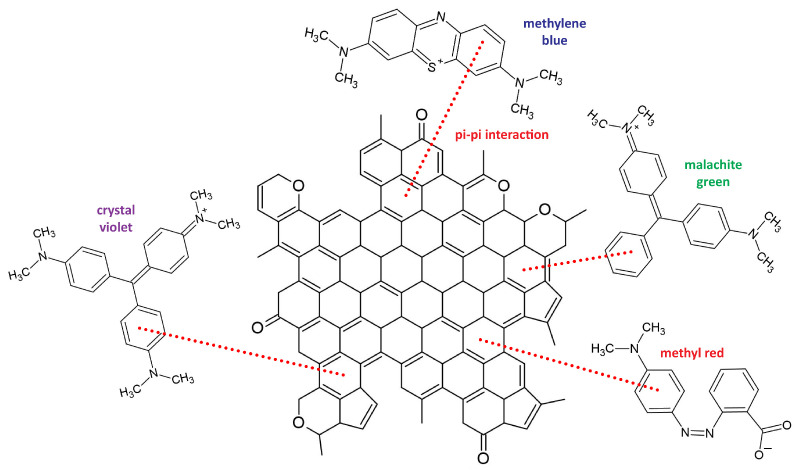
Organic dyes adsorption on activated carbons.

We also made an attempt at the determination of the pH of the obtained carbon adsorbents, in the range from 2 to 12, by the drift method. In this pH range only for sample PC5A7 it was possible to find the value of pH_pzc_, equal to 6.1. However, as mentioned above, the activated carbon samples studied have only basic functional groups on their surfaces, which means that upon adsorption, electrostatic interactions between the adsorbent surface and adsorbate molecules either do not occur or their role in the mechanism of adsorption is insignificant. It is reasonable to suppose that the effect of π-π stacking interactions between the activated carbons structure and the dye molecules may be high (Figure 7). Detail explanation of the mechanism of the dye adsorption on the obtained carbon materials requires further studies.

The parameters for the pseudo-first and pseudo-second-order models are collected in Table 6. According to these data, the kinetics of adsorption of organic dyes on the carbon materials studied is better described by the pseudo-second-order model. This is evidenced by the fact that the sorption capacities of the carbon adsorbents (q_e_) were similar to the theoretically predicted value of q_e,cal_ and the R^2^ values for this model were closer to unity.

Some of the maximum adsorption capacities (Table 2, Table 3, Table 4 and Table 5) obtained for the samples studied in this work were comparable to or higher than the literature values reported for the adsorbents obtained from different precursors (Table 7). For example, the adsorption capacities towards methyl red exceed the results obtained for inorganic adsorbents (e.g., silica-coated magnetic nanoparticles) [32], although they are much lower than the results obtained for the carbon samples obtained by chemical activation of cassava peels by H_3_PO_4_ (optimum adsorption capacity 206.08 mg/g) [33]. Similar results were obtained for methylene blue. For example, the adsorption capacities of the activated carbon samples studied are higher than those for chitosan–montmorillonite/polyaniline nanocomposite [34] and commercial activated carbon CWZ-22, but lower than for NORIT SX2 [35]. The adsorption capacities towards malachite green exceeded those for the composites of cellulose nanofiber and silver nanoparticles [36], reduced graphene oxide [37], and carbon obtained from date stones [38]. It should be also mentioned that the costs of adsorbents described in [36,37,38] are by far higher than those of obtaining activated carbons from marigold flowers. The carbon samples obtained by us showed much lower adsorption capacities towards crystal violet than the adsorbents obtained from agricultural rice bran waste [39]. However, sample PC5A8 was found to be a much more effective adsorbent of this dye than the cross-linked chitosan-coated bentonite [40].

Comparing the results obtained by our group with those reported by other research groups it should be emphasized that the activated carbons obtained from the residue of supercritical extraction of marigold flowers were characterized as adsorbents of four organic dyes of different structures and properties, whereas the majority of adsorbents described in the literature (Table 7) have been tested for the removal of only one dye. The activated carbons obtained and characterized by us may be dedicated to a much wider gamut of organic dyes, which is expected to lead in the future to a shortening of the time of adsorption and reduction of the cost of the process.

### 3.2. The Effect of Physicochemical Properties on the Adsorption of Organic Dyes

In order to elucidate or better understand the adsorption mechanism, it is necessary to consider the adsorbent surface properties. That is why, we analyzed the relations between the adsorption capacities of the samples studied and the content of the mineral substance in them, their textural parameters, surface pH, and the content of oxygen functional groups.

**Figure 8:**
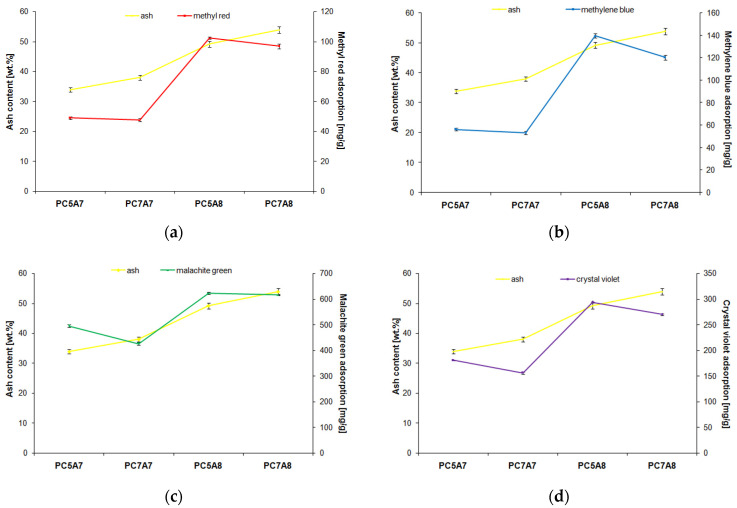
Dependence of carbon adsorbent s adsorption capacity on the mineral matter content for adsorption of: (**a**) methyl red, (**b**) methylene blue, (**c**) malachite green, (**d**) crystal violet.

According to the data from Figure 8, the content of mineral substance (ash) has a positive effect on the removal of organic dyes from water solutions as much higher sorption capacities for all four organic dyes were noted for the samples activated at 800 °C, in particular sample PC7A8 showed much higher ash content than samples PC5A7 and PC7A7.

However, a too high content of ash (about 60 wt. % in sample PC7A8) becomes unwanted ballast responsible for the fact that the adsorption capacity of sample PC7A8 is lower than that of PC5A8 by a few—for methyl red and methylene blue and even a few tens mg/g for malachite green and crystal violet.

**Figure 9:**
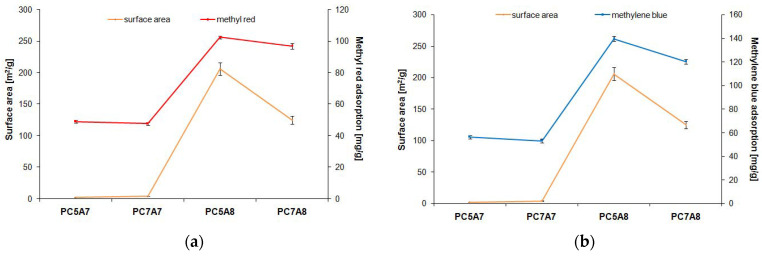

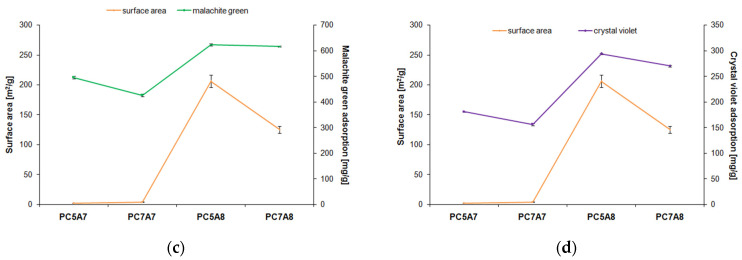
Dependence of the carbon adsorbents adsorption capacity on their surface area for adsorption of: (**a**) methyl red, (**b**) methylene blue, (**c**) malachite green, (**d**) crystal violet.

**Figure 10:**
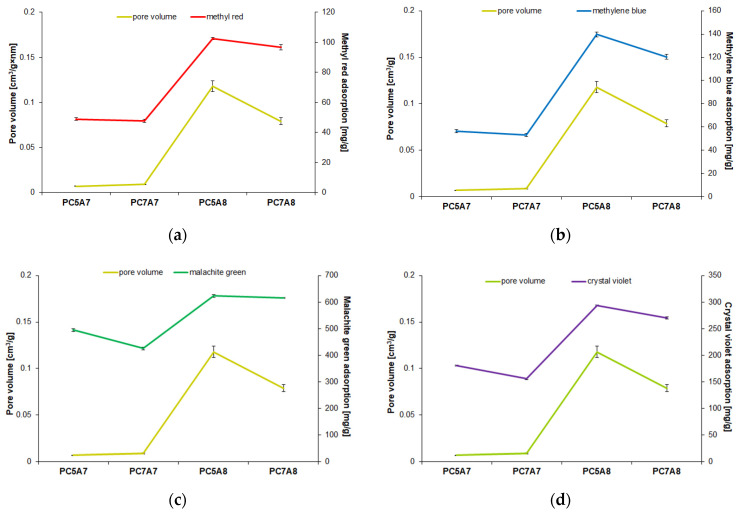
Dependence of the carbon adsorbents adsorption capacity on their total pore volume for adsorption of: (**a**) methyl red, (**b**) methylene blue, (**c**) malachite green, (**d**) crystal violet.

**Figure 11:**
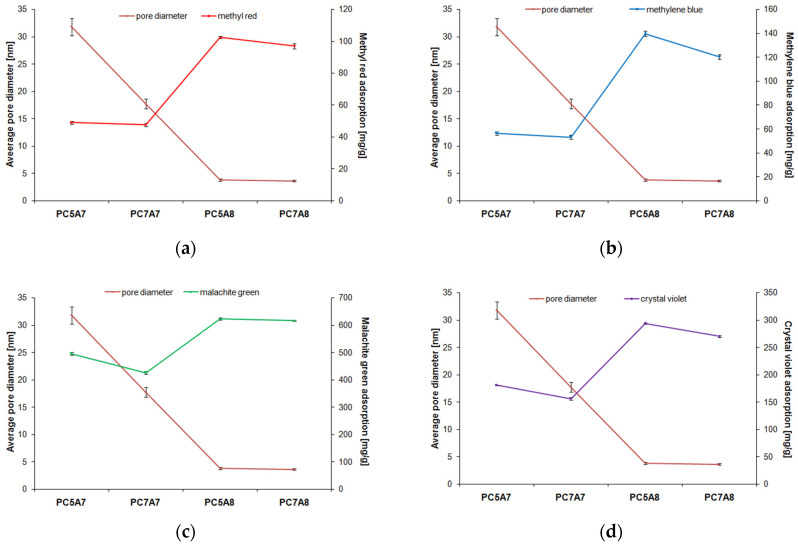
Dependence of the carbon adsorbents adsorption capacity on their average pore diameter for adsorption of: (**a**) methyl red, (**b**) methylene blue, (**c**) malachite green, (**d**) crystal violet.

Figure 9, Figure 10 and Figure 11 present the relationship between the adsorption capacities and textural parameters of the activated carbon samples studied. The porous structure of activated carbons was characterized on the basis of low-temperature adsorption/ desorption of nitrogen, using an Autosorb iQ analyzer made by Quantachrome (Boynton Beach, FL, USA). The surface area of the samples was determined by the BET (Brunauer-Emmett-Teller) method, while the pore size distribution and pore volume were calculated on the basis of the BJH (Barrett-Joyner-Halenda) model.

**Figure 12:**
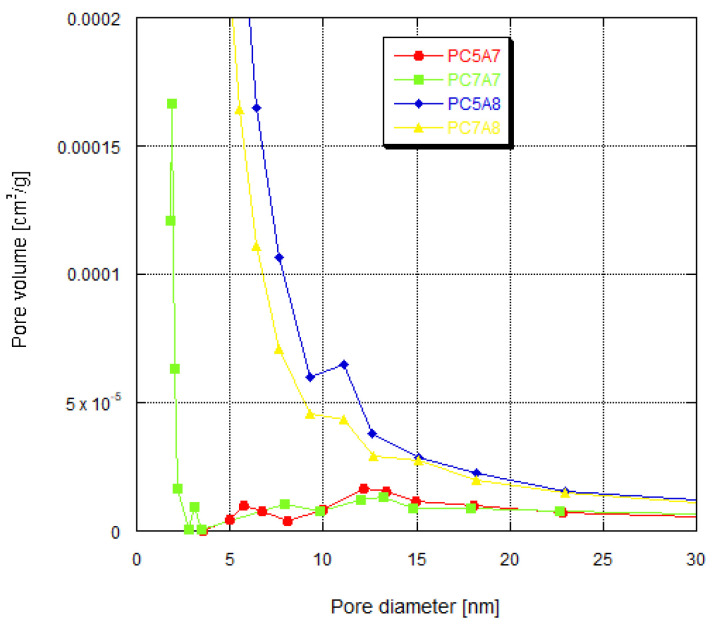
Pore size distribution for activated carbons prepared.

As indicated by Figure 9 and Figure 10, the stronger the developed surface area and porous structure, the higher the adsorption capacities. This relation is particularly well pronounced for methyl red, methylene blue, and crystal violet for which the adsorption capacity of samples PC5A8 and PC7A8 (of larger surface areas and better developed porous structure) is over twice greater than for samples PC5A7 and PC7A7. The reason for such significant differences in the adsorption capacities of the carbon samples activated at 700 (A7) and 800 °C (A8) can also be a different character of the porous structure. According to the data presented in Figure 11, the samples PC5A7 and PC7A7 have mainly large pores of an average pore diameter of 33 and 17 nm, while the samples activated at 800 °C have pores of an average diameter of about 4 nm. Moreover, as follows from the character of PSD curves presented in Figure 12, samples PC5A8 and PC7A8 contain a great number of mesopores of diameters from 5 to 20 nm, whose presence is conducive to adsorption of molecules of the sizes close to those of the organic dyes studied.

**Figure 13:**
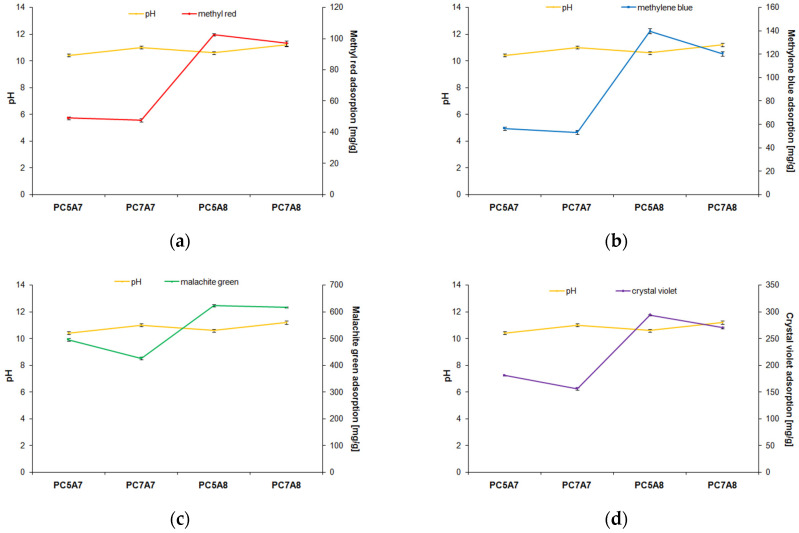
Dependence of dyes adsorption capacity on surface pH value: (**a**) methyl red, (**b**) methylene blue, (**c**) malachite green, (**d**) crystal violet.

**Figure 14:**
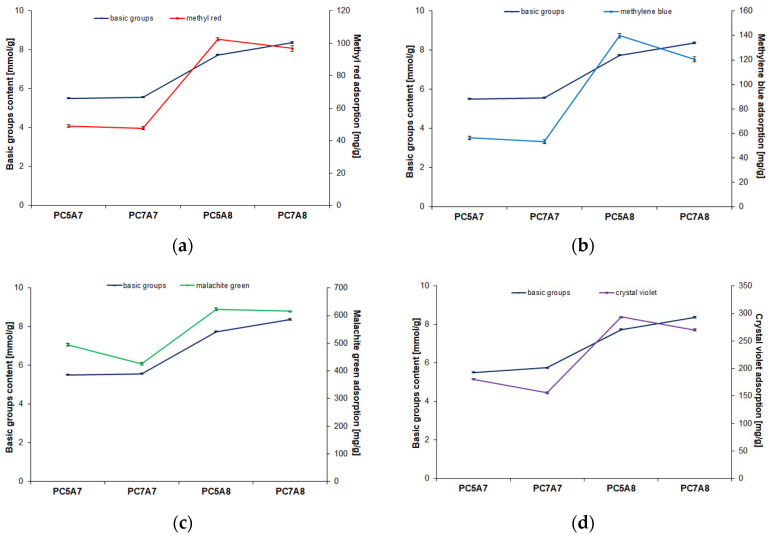
Dependence of dyes adsorption capacity on basic groups content: (**a**) methyl red, (**b**) methylene blue, (**c**) malachite green, (**d**) crystal violet.

The chemical character of the activated carbon adsorbents also has some influence on the adsorption capacities. Although no correlation between the surface pH and adsorption capacities is found, see Figure 13, there is a significant correlation between the content of basic functional groups on the sample’s surface and the effectiveness of the organic dyes adsorption, (Figure 14). The samples PC5A8 and PC7A8 showing high contents of basic groups on the surfaces have much higher adsorption capacities towards all the dyes studied. It should be added that the activated carbon samples studied did not contain acidic functional groups.

## 4. Conclusions and Future Perspectives

The above presented and discussed results have shown that the residues of supercritical extraction of marigold can be used for the production of activated carbon adsorbents characterized by high effectiveness in removal of organic dyes from water solutions. It should be emphasized that the samples obtained in this way show not only high effectiveness of adsorption of liquid impurities, but are also efficient in the removal of pollutants from the gas phase, which illustrates the wide range of their application. The adsorption properties of the activated carbons studied are comparable to or even better than those of the commercial adsorbents and other ones described in the literature. It should be also mentioned that the carbon adsorbents described by other authors have been usually obtained by chemical activation with the use of expensive activators, which substantially increases the cost of their production. The samples obtained and characterized by us were obtained by physical activation, which was much cheaper and permitted obtaining high sorption capacities.

The most effective adsorbent of the dyes studied was sample PC5A8, obtained by physical activation at 800 °C of carbonizate PC5. According to the results, although the activated carbon adsorbents obtained have poorly developed surface areas, they show high adsorption capacities towards the organic dyes of different molecular sizes. The surface area is not the only parameter determining the sorption capacities of the samples studied as these properties also depend on the content of ash and the presence of surface oxygen functional groups.

On the basis of the parameters of Langmuir, Freundlich, and Temkin adsorption isotherms, the adsorption on the carbon samples studied was found to follow the Langmuir model, which implies the formation of a monolayer of adsorbed substance on the adsorbent surface. The adsorption capacities of the activated carbon samples studied increase with increasing initial concentration of the organic dye in the solvent, which suggests that at low dye concentrations their adsorption has a random character, while at high concentrations the active centers on the adsorbent’s surface are all occupied and the adsorbent is saturated. The kinetics of adsorption of organic dyes was found to be described by the pseudo-second-order model. The results indicate that further studies on the use of the residues of supercritical extraction of plants should be directed at obtaining adsorbents of stronger developed surface area and with a greater contribution of small mesopores in the total pore volume, showing a high content of basic functional groups on the surface and a relatively high content of mineral substances. Another important problem that needs explanation is the mechanism of the reactions between the adsorbents and adsorbates. Detail examination of this mechanism will permit obtaining adsorbents dedicated to simultaneous and effective removal of a wide gamut of organic pollutants.

## Figures and Tables

**Table 1:** Textural parameters obtained activated carbons compared with other adsorbents.

Sample	Surface Area [m^2^/g]	Pore Volume [cm^3^/g]	Pore Size [nm]	References
PC5A7	2	0.007	31.78	[21], this study
PC7A7	4	0.009	17.71	[21], this study
PC5A8	206	0.118	3.79	[21], this study
PC7A8	125	0.079	3.63	[21], this study
chamomile	64	0.060	3.71	[27]
hops	75	0.040	11.74	[9]
Sapelli sawdust	300	0.166	2.2	[28]
coconut shell	437	0.21	3.53	[29]
Norit^®^ SX ULTRA	1092	1.05	4.4	[30]

**Table 2:** Langmuir, Freundlich and Temkin parameters of the adsorption isotherms of organic dyes onto activated carbon—PC5A7.

Dye	C_0_ [mg/L]	R [%]	q_e_ [mg/g]	Langmuir				Freundlich			Temkin		
				R^2^	q_max_ [mg/g]	K_L_ [L/mg]	R_L_	R^2^	K_F_ [mg/g(L/mg)^1/n^]	n	A_T_ [L/mg]	B	R^2^
MR	5–35	100–71	48.95 ± 1.00	0.9903	49.02	0.043	0.82–0.39	0.9753	27.06	3.7175	2.71	15.63	0.7608
MB	5–40	100–73	56.36 ± 1.38	0.9938	56.82	0.072	0.74–0.29	0.9963	38.36	5.7670	1.43	7.62	0.9131
MG	5–260	100–97	495.04 ± 5.00	0.9673	526.32	0.002	0.58–0.32	0.8547	262.24	24.5700	7.61	121.37	0.9550
CR	5–100	100–90	180.68 ± 0.69	0.9871	181.82	0.013	0.80–0.57	0.9753	111.87	4.6707	5.81	45.77	0.5989

**Table 3:** Langmuir, Freundlich and Temkin parameters of the adsorption isotherms of organic dyes onto activated carbon—PC7A7.

Dye	C_0_ [mg/L]	R [%]	q_e_ [mg/g]	Langmuir				Freundlich			Temkin		
				R^2^	q_max_ [mg/g]	K_L_ [L/mg]	R_L_	R^2^	K_F_ [mg/g(L/mg)^1/n^]	n	A [L/mg]	B	R^2^
MR	5–35	100–68	47.62 ± 1.02	0.9964	47.85	0.068	0.74–0.29	0.9963	29.42	4.3898	4.54	11.50	0.9949
MB	5–40	100–70	53.14 ± 1.51	0.9994	53.19	0.221	0.31–0.10	0.8184	43.80	11.5607	1.78	7.21	0.8696
MG	5–230	100–94	425.46 ± 5.01	0.9872	434.78	0.003	0.76–0.58	0.9690	243.05	5.9488	21.02	77.72	0.9846
CR	5–90	100–89	155.91 ± 1.71	0.9864	156.25	0.017	0.59–0.34	0.9659	94.19	4.3234	3..37	48.30	0.6889

**Table 4:** Langmuir, Freundlich and Temkin parameters of the adsorption isotherms of organic dyes onto activated carbon—PC5A8.

Dye	C_0_ [mg/L]	R [%]	q_e_ [mg/g]	Langmuir				Freundlich			Temkin		
				R^2^	q_max_ [mg/g]	K_L_ [L/mg]	R_L_	R^2^	K_F_ [mg/g(L/mg)^1/n^]	n	A [L/mg]	B	R^2^
MR	5–60	100–85	102.43 ± 0.79	0.9954	104.17	0.035	0.58–0.32	0.8547	73.06	5.6180	4.54	31.43	0.5602
MB	5–80	100–87	139.72 ± 2.04	0.9983	140.85	0.036	0.41–0.26	0.9109	107.03	8.0128	6.43	16.28	0.7359
MG	5–330	100–97	622.80 ± 5.16	0.9969	625.00	0.026	0.13–0.10	0.7491	467.74	8.0972	27.45	113.87	0.7296
CR	5–160	100–93	293.75 ± 0.88	0.9957	294.12	0.012	0.46–0.35	0.9609	211.98	7.8125	8.56	68.85	0.4910

**Table 5:** Langmuir, Freundlich and Temkin parameters of the adsorption isotherms of organic dyes onto activated carbon—PC7A8.

Dye	C_0_ [mg/L]	R [%]	q_e_ [mg/g]	Langmuir				Freundlich			Temkin		
				R^2^	q_max_ [mg/g]	K_L_ [L/mg]	R_L_	R^2^	K_F_ [mg/g(L/mg)^1/n^]	n	A [L/mg]	B	R^2^
MR	5–60	100–82	96.88 ± 1.77	0.9757	100.00	0.012	0.80–0.57	0.9753	48.74	3.2144	2.98	29.96	0.8067
MB	5–70	100–88	120.39 ± 2.01	0.9930	120.48	0.115	0.18–0.11	0.9176	107.18	19.1204	5.71	15.96	0.6579
MG	5–330	100–97	615.96 ± 2.07	0.9917	625.00	0.004	0.73–0.46	0.9839	412.38	11.3250	71.18	92.64	0.9796
CR	5–150	100–93	269.99 ± 2.00	0.9979	270.27	0.020	0.34–0.25	0.9435	212.67	9.6154	8.35	65.40	0.4556

**Table 6:** Adsorption kinetics parameters for the adsorption of organic dyes onto activated carbons.

Sample	Dye	q_e_ [mg/g]	Pseudo-First Order Model			Pseudo-Second Order Model		
			R^2^	k_1_ [L/min]	q_e,cal_ [mg/g]	R^2^	k_2_ [g/mg·min]	q_e,cal_ [mg/g]
PC5A7	MR	41.03 ± 0.82	0.9028	4.61 × 10^−3^	17.73	0.9996	2.33 × 10^−3^	41.32
PC7A7	MR	42.00 ± 0.76	0.9983	5.52 × 10^−3^	18.36	0.9996	1.98 × 10^−3^	42.55
PC5A8	MR	57.35 ± 1.38	0.9969	1.27 × 10^−2^	17.72	0.9998	2.22 × 10^−3^	58.14
PC7A8	MR	57.40 ± 0.80	0.9957	1.24 × 10^−2^	17.68	0.9997	2.21 × 10^−3^	58.82
PC5A7	MB	50.29 ± 0.96	0.9589	8.98 × 10^−3^	33.82	0.9967	6.18 × 10^−4^	52.63
PC7A7	MB	50.05 ± 0.75	0.9714	7.83 × 10^−3^	34.78	0.9968	6.17 × 10^−4^	52.61
PC5A8	MB	59.62 ± 1.31	0.8619	3.75 × 10^−2^	31.16	0.9999	6.22 × 10^−3^	60.24
PC7A8	MB	59.98 ± 1.08	0.9532	7.14 × 10^−3^	13.84	0.9998	2.10 × 10^−3^	60.01
PC5A7	MG	380.63 ± 4.19	0.9669	8.29 × 10^−3^	39.33	0.9999	8.58 × 10^−3^	381.68
PC7A7	MG	383.23 ± 4.98	0.9812	7.60 × 10^−3^	20.80	0.9999	9.81 × 10^−3^	384.62
PC5A8	MG	400.00 ± 8.80	0.9949	7.83 × 10^−2^	20.14	0.9999	2.32 × 10^−3^	400.00
PC7A8	MG	383.03 ±7.28	0.8285	2.41 × 10^−2^	14.27	0.9999	8.52 × 10^−3^	383.14
PC5A7	CR	168.34 ± 3.87	0.9628	3.64 × 10^−2^	33.45	0.9999	2.07 × 10^−3^	175.44
PC7A7	CR	155.91 ± 3.27	0.9870	6.45 × 10^−3^	22.40	0.9999	2.61 × 10^−3^	156.25
PC5A8	CR	180.00 ± 2.88	0.9959	8.06 × 10^−3^	22.48	0.9999	2.26 × 10^−3^	178.52
PC7A8	CR	180.00 ± 3.60	0.9478	9.68 × 10^−3^	20.05	0.9999	2.39 × 10^−3^	181.82

**Table 7:** Adsorption capacity of selected materials.

Material	Dye	Maximum Capacity [mg/g]	Article
marigold	methyl red	102.43	(this study)
silica coated magnetic nanoparticles		49.50	[32]
cassava peels		206.08	[33]
charred sawdust		70.0	[4]
marigold	methylene blue	139.72	(this study)
chitosan–montmorillonite/polyaniline nanocomposite		111	[34]
CWZ-22		130	[35]
NORIT SX2		150	[35]
marigold	malachite green	622.80	(this study)
composites of cellulose nanofiber and silver nanoparticles		142	[36]
date stones		98	[37]
reduced graphene oxide		279.85	[38]
marigold	crystal violet	293.75	(this study)
agricultural rice bran waste		603.00	[39]
cross-linked chitosan coated bentonite.		169.49	[40]

## Data Availability

Data are contained within the article.
